# Does Digitally Enabling Frontline Health Workers Improve Coverage and Quality of Maternal and Child Health Services? Findings From a Mixed Methods Evaluation of TECHO+ in Gujarat

**DOI:** 10.3389/fpubh.2022.856561

**Published:** 2022-07-22

**Authors:** Somen Saha, Zahiruddin Syed Quazi

**Affiliations:** ^1^Indian Institute of Public Health Gandhinagar (IIPHG), Gandhinagar, India; ^2^Jawaharlal Nehru Medical College, Datta Meghe Institute of Medical Sciences, Wardha, India

**Keywords:** mHealth, mother and child care, Gujarat, technology, Management Information System (MIS)

## Abstract

**Introduction:**

Technology Enabled Community Health Operations (TeCHO+) is a mobile and web-based application (app) for frontline health workers. It includes features such as real-time data entry, automated generation of the work plan, and a decision support system generating alerts for high-risk cases. Since 2019, the programme is implemented across all 33 districts of Gujarat, catering to a population of over 60 million. This study aims to compare changes in the coverage, quality of data reporting maternal and child health services, and time spent in the documentation before and after the introduction of the TeCHO+ app.

**Methods:**

To address the study aim, a mixed-method design with a realist evaluation approach was adopted. The survey was conducted with randomly selected beneficiaries from 32 sub-centers across two districts of Gujarat State in India. We surveyed 215 postpartum women and mothers of 102 children at baseline (pre) and 246 postpartum women and mothers of 119 children post 1 year of the TeCHO+ programme intervention in 2020. For qualitative data, total 29 Auxiliary Nurse Midwives, 12 Data Entry Operators and 10 Primary Health Center Medical Officers were purposively selected from 32 PHCs and interviewed to understand the pathways leading to the programme outcome.

**Results:**

Following introduction of TeCHO+, the coverage of full antenatal care (ANC; 75.6% vs. 67.9%, *p*-value < 0.0001), consumption of at least 180 iron-folic acid tablets (93% vs. 77%, *p*-value < 0.001), early initiation of breastfeeding (42.7% vs. 24.2%, *p*-value < 0.001), five home-visits by ANM during the first month after delivery (36.2% vs. 27.9%, *p*-value = 0.056), HBV0 vaccination (67.2% vs. 35.3%, *p*-value < 0.0001) and Pentavalent 2 (100% vs. 95.1%, *p*-value = 0.015) improved. The overall concordance rate for routine maternal health indicators (a measure of data quality) improved from 69.1 to 80.5%, while that for routine child health indicators improved from 86.6 to 92.1%. The programme resulted in 1.7 h saving a day of ANM's productive time and 1.5 h (a day) of data entry operator's time.

**Conclusions:**

The TeCHO+ programme has improved access to care. It impacted both coverage of maternal and child health services and data reporting quality of various maternal and child high-risk conditions. Considering the programme's success, other disease services might be added to the scope of TeCHO+ software.

## Strength and Limitations of the Study

- Evidence on the use of mHealth technology solutions by frontline health workers in controlled settings is promising. However, the studies on the effectiveness of the large-scale implementation of mHealth interventions are limited.
- TeCHO+ programme is one such mHealth programme implemented in the entire state of Gujarat.
- Qualitative findings indicate more opportunities than challenges in implementation of TeCHO+ programme, noted improvements in service coverage, quality of data reporting and found to be time-saving. It saved time of auxiliary nurse midwives (1.7 h a day) and data entry operators (1.5 h a day).
- TeCHO+ programme has the potential to improve the quality of data reporting and also improving the coverage of healthcare services and should be considered for scale-up.

## Introduction

Coverage of maternal and child health (MNCH) services continues to remain a cause of concern. In Gujarat, one of the western states of India, only 30.7% of the pregnant women received full ante-natal care (ANC), and 50.4% of the children were fully immunized in 2015–2016, according to the National Family Health Survey-4 report (1). Enabling frontline health workers with mobile-phone-technology–based health (mHealth) solutions promises to increase effective coverage of some of the maternal and child healthcare services by improving the performance of frontline health workers in controlled settings on a limited scale (2–7). However, evidence on the impact of mHealth technology on frontline health workers has been limited to pilot-level interventions at a limited scale.

Recognizing the need to create disaggregated data for better tracking of mother and child *e-Mamta* programme was launched in 2010 under the National Rural Health Mission (NRHM) in Gujarat. The e-Mamta software was designed to register pregnant women and children from 0 to 6 years of age and to capture details on ensuring complete service delivery of antenatal Care (ANC), Child birth, Post Natal Care (PNC), Immunization, nutrition and to identify the drop-outs. Mother and child records were digitized at primary health centers by data entry operator. This created a delay in data entry, hindering real-time high-risk case management, duplications and error in reporting (8, 9). From April 2019, Technology Enabled Community Health Operations Plus (TeCHO+) programme was introduced by the Department of Health and Family Welfare, Government of Gujarat, replacing the e-Mamta programme. TeCHO+ is an upgraded version of the successful pilot – ImTeCHO implemented by the Department of Health and Family Welfare, Government of Gujarat (10). The TeCHO+ programme, a mobile and web-based application (app) is implemented across all 33 districts, catering to a population of over 60 million.

## Programme Theory

Theories depicting how and in what circumstances an intervention is proposed to change programme outcomes are known as programme theories (11). The TeCHO+ programme is expected to improve service coverage and data management. The key features and implementation context of the TeCHO+ programme are explained below.

### Unique Features of the TeCHO+

The programme encompasses unique features such as real-time data entry (online and offline) by auxiliary nurse midwives (ANM), artificial intelligence enabled tracking of beneficiaries for migration as well as utilization of maternal and child health services, tracking frontline health workers for daily login, and generation of automatic reports. One of the unique features of the TeCHO+ is an in-built algorithm that stratifies risks based on the input of beneficiaries details and notifies high-risk cases to ANMs, their respective PHC-MOs and Taluka TeCHO+ Coordinator for action. This feature encourages timely case management of high-risk cases. Further, this application pops up relevant educational videos based on risk assessment of the beneficiary to ensure correct messages are reached to the beneficiary as per the need. The TeCHO+ is also enabled with a web-based dashboard that enables health officials at different levels to access progress reports and provide supportive supervision to frontline health workers.

### Human Resource Structure

The programme has created structures for effective implementation. At the state level, the TeCHO+ task force is established to oversee the overall coordination, planning, and decision-making, chaired by the Health Commissioner of Gujarat. Nodal officer is deputed at the state level to coordinate and oversee TeCHO+ programme implementation. For technical field support, district coordinators were recruited in the TeCHO+ programme. Chief District Health Officers (CDHOs) and Reproductive and Child Health Officers (RCHOs) at the district level and, at the block level, Taluka Health Officers are entrusted with the responsibility of training, supervision, and monitoring of the programme. Furthermore, Primary Health Center-Medical Officers (PHC-MOs) are assigned the task to verify the families in case of the death of a family member or immigration or emigration of a family or a particular member of the family. Territorial TeCHO+ coordinators were recruited initially for field monitoring but discontinued from April 2020 onwards, and PHC-MOs are now doing field monitoring with the help of a web-based interface.

### Supervision, Training and Support

The Programme has robust supervision, monitoring and support mechanisms at each level. Supervisory trainings were imparted to all supervisory cadre of the TeCHO+ team. At the state level, the State TeCHO team includes an allopathic Medical Officer, State Data Manager, IT Technician, Programme Officer and Programme Assistant (deputed from the e-Mamta division). State Data Manager and Medical Officer are primarily responsible for supervision and monitoring of the TeCHO+ as well as provide feedback to software service company for modification and upgradation of the software. The state health officials use WhatsApp and SATCOM (Satellite communication facilitated by Bhaskaracharya Institute for Space Applications and Geo-Informatics – BISAG, Gandhinagar) for building the capacity of the staff. In the initial phase, territorial TeCHO coordinators were recruited who were responsible for supervising ANMs at the field level. Besides, the command and control center was established to randomly call beneficiaries to validate the scheduled service provision by ANMs.

There was a two-way – between community level and facility level – referral system as part of the TeCHO+ for better continuum of care. TeCHO+ platform was used by providers at community and facility levels. It had a common denominator of beneficiaries with each having a unique ID number. Therefore, there was data exchange between the community and facility levels. The data exchange was about variety of information including demographic information, and clinical information.

Additionally, there was a centralized call center that was using TeCHO+ platform. The call center played an important role in referral of high risk RMNCH cases. Following are some examples of two-way referral.

1. When a frontline health worker identifies a high-risk case at the community level the information of the case was displayed on the PHC/facility dashboard of TeCHO+. Subsequently, this information helped PHC to ensure referral of the case.
2. Similarly, if an event (such as delivery) is recorded by the facility then the record is updated in respective frontline health worker's TECHO+ mobile phone application automatically by the software system. Then, the community based frontline worker starts receiving the reminders for subsequent community-based care (such as postnatal care and vaccination).

### Sustainability

TeCHO+ is shared with the national RCH portal. TeCHO+ has become one of the important sources of data for project reviews on consistent basis at every level from the State, district, block and PHC levels. TeCHO+ data is fed and reviewed by the Gujarat Chief Minister's digital dashboard. In light of high adoption of TeCHO+, the Gujarat government has issued instructions that ANMs may not maintain paper registers anymore. This has been an important incentive for the ANMs to use TeCHO+ and save time for record keeping.

As the aim of the TeCHO+ programme stated to be improving service coverage and data quality, the purpose of the present study was to evaluate whether the TeCHO+ programme succeeded in its aims. Using a realist evaluation framework, the study findings are expected to generate the evidence needed to inform decisions pertaining to the continuation of the TeCHO+ programme in the state and inform possible nationwide scale-up.

## Materials and Methods

### Study Design and Study Setting

As TeCHO+ is implemented in the entire state, it was not possible to find a comparison district. Thus, for evaluating the impact of the TeCHO+ programme, a single group, pre and post intervention design was the most appropriate study design for gathering quantitative data. We used mixed methods combining quantitative and qualitative research methodology using a realist evaluation framework to assess programme outcomes as well as contextual factors that influence programme outcomes. Realist evaluation is an explanation-driven form of research inquiry guided by critical realism (12). Realist evaluation considers that programmes are complex interventions introduced into social systems, which are complex indeed (13). Depending on the context, interventions offer the resources to the recipient, to which the recipients may or may not respond. The performance of the intervention is contingent not only on the nature of interventions alone but also on how recipients respond to the resources offered by the intervention (13). Thus, realist evaluation elucidates contextual influences and meanings that help explain factors that make it work (14).

The data was collected from two districts of Gujarat viz. Devbhumi Dwarka and Panchmahal using survey tool and semi-structured interviews by trained data collectors. The detailed protocol titled “TeCHO+ program in Gujarat: a protocol for health technology assessment” has been reported elsewhere (15).

### Intervention

The TeCHO+ programme is a mobile and web-based application that enables data entry by frontline health workers – Auxiliary Nurse Midwifery (ANM) providing services at the time and place of health service delivery to improve decision-support in delivering quality maternal and child healthcare. Automated generation of daily work-plan capacitates the ANMs to track beneficiaries, and alerts for high-risk cases help ANMs follow-up and encourage beneficiaries to take referral services. The web-based dashboard enables health officials at different levels to access progress reports and extends supportive supervision to ANMs.

### Comparator

The e-Mamta software is a name-based tracking system for mothers and children. The e-Mamta health information software was designed to register pregnant women and children and report various maternal and child health indicators. The data pertaining to the registration of new beneficiaries and provision of services to the existing beneficiaries was maintained by the ANMs in registers at their respective sub-centers. The data was then shared with the PHCs, where a dedicated Data Entry Operators (DEOs) enter the data into the e-Mamta portal. Based on the data entered, the software helped generate the work plan for ANMs. The software also had the feature which enabled the supervisory staff to monitor the service delivery.

### Outcomes and Their Measurement

The study team undertook the validation exercise in sampled households of the above mentioned districts wherein the data was collected to verify the quality of data and coverage of services. For establishing the baseline, data of the sampled households were retrieved from the e-Mamta software (March 2019). This data was validated through a field survey. One year after the programme implementation (February 2020), data were retrieved from the TeCHO+ software. In addition, a validation study of the TeCHO+ programme was undertaken for morbidity related indicators among high-risk women and children. Details of their maternal and child health services were compared with the field survey. Since we intended to evaluate the TeCHO+ software with e-Mamta as the comparator, the common indicators of both these routine health information systems (RHIS) were selected for this study. The indicators used for the evaluation study are mentioned in Supplementary Table 1.

We considered Full ANC amongst pregnant women, as those women who undergone four antenatal check-ups, tetanus toxoid (TT) vaccine and consumed 180 iron folic acid (IFA) tablets. A child was considered to be fully immunized if he/she received one dose of Bacillus Calmette Guerin (BCG), three doses of diphtheria, pertussis and tetanus (DPT), three doses of polio vaccines, and one dose of measles vaccination by the age of 9–12 months.

### Participants' Inclusion Criteria

The inclusion criteria were:

(1) Women who have delivered between 1st November 2018 to 31st January 2019 for baseline and between 1st October 2019 to 31st December 2019 for follow-up of the survey.
(2) Children in the age group of 12–15 months for child-related indicators, i.e. (1st November 2017 to 31st January 2018 for baseline and 1st October 2018 to 31st December 2018 for follow-up survey); and
(3) Pregnant women and children with specific high-risk conditions (2019).

### Participants Exclusion Criteria for Validation

For the proposed study, the population residing in urban talukas and municipal corporation areas were not included.

### Sampling and Sample Size

**Figure 1:**
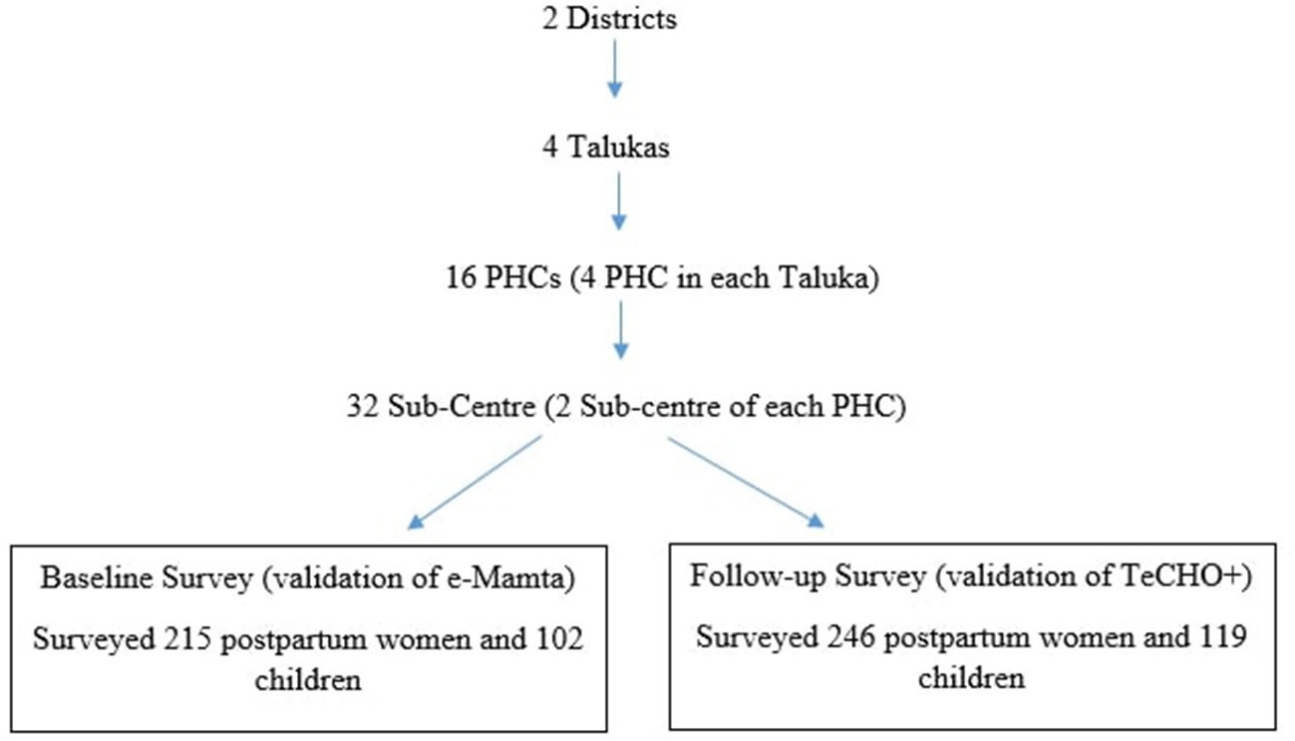
Sampling of the study.

The study was conducted from beneficiaries selected from 32 sub-centers spread across two selected districts of Gujarat. The selection of Talukas was done purposively based on their distance to their respective headquarters (two talukas, i.e., one nearest and one farthest from the district headquarters, were selected). However, a simple random sampling method was adopted to select the PHC and Sub-Center using the table of random numbers. The flow chart presents the sampling of the proposed study (Figure 1). Universal sampling was adopted to select the beneficiaries adhering to the inclusion and exclusion criteria and providing consent for their participation. We surveyed 215 postpartum women and mothers of 102 children at baseline and 246 postpartum women and mothers of 119 children after 1 year of programme intervention in 2020. The detailed study protocol was published elsewhere (15). Although the original study was planned across five districts of Gujarat, due to COVID-19 associated restriction, the final study was conducted among samples from 2 districts of Gujarat.

Moreover, for morbidity and its management related indicators, all the high-risk women suffering from severe maternal anemia and pregnancy-induced hypertension and children suffering from severe acute malnutrition and low birth weight reported at the selected sub-centers were surveyed in 2020.

### Assessing Pathways to the Observed Programme Outcomes

Several factors are expected to contribute to the observed effect. We performed a participatory process documentation study with the key stakeholders involved in the roll-out of the TeCHO+ programme to understand the early implementation experience of the TeCHO+ programme, which was published elsewhere (16). Through the participatory process documentation, we gained an in-depth understanding of the contextual factors, including software application, supportive supervision, behavior changes among ANMs and Primary Health Center Medical Officers (PHC-MOs) in implementing the programme. This followed the standard realist proposition to explore the contextual conditioning.

A qualitative method was adopted to understand the contribution of each of the programme components. The themes that emerged while documenting the early implementation experience of the TeCHO+ programme provided the framework for designing the semi-structured interview guides. Semi-structured interviews were used to gather responses of the field level staff directly responsible for implementing the TeCHO+ programme and estimate the time spent by the primary users of e-Mamta and TeCHO+ software. Field level staff included ANM, data entry operators (DEOs) who were managing the data entry in eMamta before the launch of TeCHO+ and 10 PHC-MOs. A total of 51 field level staff (29 ANMs; 12 DEOs, and 10 PHC-MOs) were purposively selected from 32 PHCs and interviewed. Interview guides were designed to solicitate conditions or contexts under which the programme operated and the patterns of outcome. Training was imparted to interviewers on detail understanding of the interview guides including exploring the advantage of using realist methodology to go beyond the question of “did an intervention work or not?”. Responses were coded into themes in terms of context, intervention elements and outcomes.

Time saved was calculated by considering the time spent for data entry of new and old ANC/PNC and Child services for 24 working days in the TeCHO+ software. For computing, the time spent on data management in e-Mamta software, data spent on daily entry of new and old ANC/PNC and child services, and daily and monthly report generation were gathered from DEO, and the time ANM used to spend toward maintaining physical registers were considered. Based on the responses, the average time for data management in TeCHO+ and e-Mamta software was computed.

### Analysis

Quantitative data was collected using an open data kit (ODK) to assess coverage as well as quality of data reporting of maternal and child health indicators. The data collectors were provided 2-day training to use the data collection tool. Descriptive analysis, as well as Pearson's chi square test, were performed on the categorical data to analyse significant difference in coverage of indicators at the baseline and follow-up survey. While for interpreting the improvement in quality of data reporting concordance rates were calculated.

For qualitative data, thematic analysis techniques using manual methods consistent with the recommendations of Strauss and Corbin (17) were adopted for transcribing, de-identifying and coding data. These techniques allowed identification of common themes within the qualitative data, without the constraint of establishing how themes linked together.

### Ethical Review

The approval to conduct the study was obtained from the Technical Appraisal Committee, HTAIn, Department of Health Research, India and by the Institutional Ethics Committee of the Indian Institute of Public Health Gandhinagar. The study participants were included based on their willingness and their readiness to provide written informed consent.

### Patient and Public Involvement

Patients or the public were not involved in the design, or conduct, or reporting, or dissemination plans of our research.

## Results

### Key Findings of the Evaluation Study

#### Quality of Data Reporting and Coverage of Routine Maternal Health Indicators

We surveyed a total of 215 postpartum women at the baseline of which we could retract the data of only 188 women from the e-Mamta software. During the follow-up survey, we approached 246 postpartum women and could retrieve details of 229 women from TeCHO+ software.

**Figure 2:**
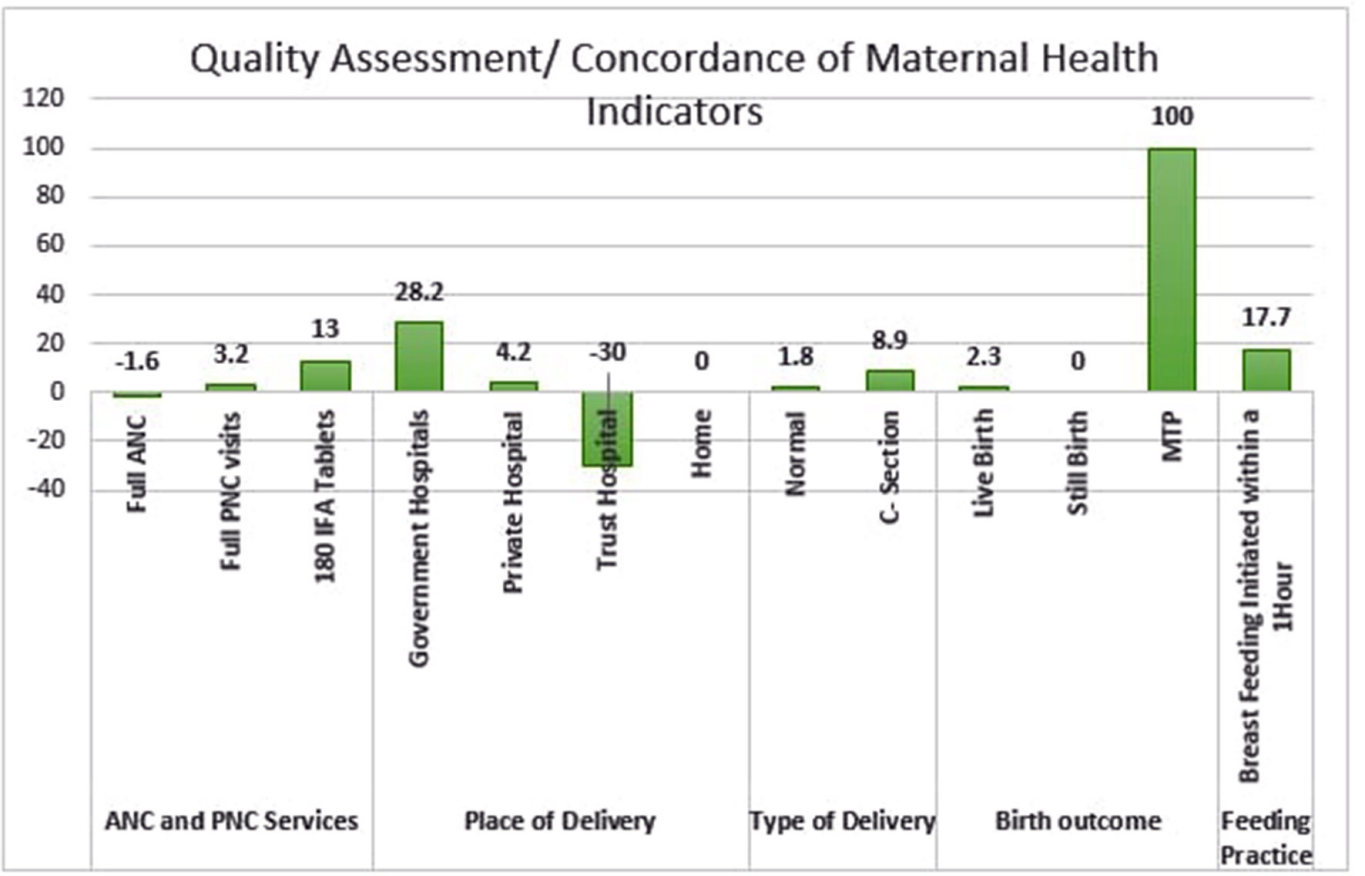
Quality assessment of maternal health indicators.

Quality of data reporting in follow-up survey improved as compared to baseline for all the indicators except for full ANC and reporting of delivery in trust hospitals. Major improvements are noted in the consumption of iron-folic acid (IFA) tablets, delivery reported in government hospitals, medical termination of pregnancy, and early breastfeeding initiation. The concordance rate for routine maternal health indicators (a measure of data quality) improved from 69.1 to 80.5% (Figure 2).

Coverage of all the maternal health indicators have improved in the follow-up survey (Table 1). There is marked improvement specifically in the consumption of 180 IFA tablets (16.3% increase in coverage) and breastfeeding initiation within an hour of birth (18.5% increase in coverage). Between the two surveys, the decline was observed in the delivery at the private and trust hospitals, cesarean section deliveries and stillbirth.

**Table 1:** Coverage of various maternal health indicators.

**Maternal health indicators**				
**Variable**	**Follow-up survey** **(*****N*** **=** **246)**	**Baseline field survey** **(*****N*** **=** **215)**	**% change in coverage between follow-up and baseline field survey**	***p*** **-value**
Full ANC visits	80.1	76.3	3.8	0.024
Full PNC	36.2	27.9	8.3	0.058
180 IFA tablets	93.5	77.2	16.3	<0.0001
**Delivery place**
Government hospitals	34.1	26	8.1	0.045
Private hospital	60.2	66.5	−6.3	
Trust hospital	0.8	3.7	−2.9	
Home	4.9	3.7	1.2	
**Type of delivery**
Normal	83.7	80.9	2.8	0.084
C-section	14.6	19.1	−4.5	
**Delivery outcome**
Live birth	98.8	98.6	0.2	0.219
Still birth	0	0.9	−0.9	
MTP	1.2	0.5	0.7	
Breast feeding initiated within a 1 h	42.7	24.2	18.5	<0.0001

Pearson Chi-square test was applied to assess the association between the change in coverage of various maternal health services. The improvement in coverage of important health indicators such as full ANC examination (80.1% vs. 76.3%, *p*-value = < 0.0001), consumption of at least 180 iron-folic acid tablets (93.5 % vs. 77.2 %, *p*-value < 0.0001), and early initiation of breastfeeding (42.7% vs. 24.2%, *p*-value < 0.001) were found to be statistically significant at 5% level of significant and 95% Confidence Interval.

#### Quality of Data Reporting and Coverage of Routine Child Health Indicators

For assessment of child health indicators, our sample constituted of 12–15 months of children. At the baseline, we surveyed 102 children, and during the follow-up survey, 119 children were interviewed. Details of 93 children and 105 children were found from e-Mamta and TeCHO+ software, respectively.

**Figure 3:**
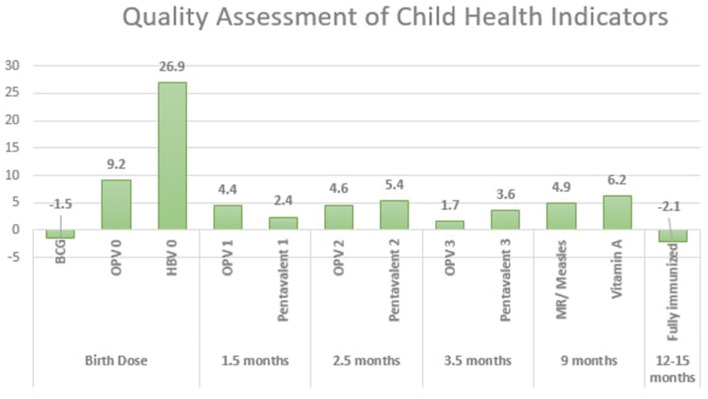
Quality assessment of child health indicators.

Improvement in the quality of data reporting were observed for almost all the child health indicators during the follow-up survey (Figure 3). The concordance rate for routine child health indicators improved from 86.6 to 92.1%. However, a marginal decline of quality of reporting was found for BCG vaccination at birth (change in concordance from 96.7 to 95.2 at follow-up) and full immunization (change in concordance from 89.6 to 87.5 at follow-up).

Coverage of immunization has increased significantly in providing HBV0 vaccine and OPV0 vaccine (Table 2). However, full immunization coverage in children of 12–15 months of age shows a non-significant decline of 10% compared to the baseline survey findings.

**Table 2:** Coverage of child health indicators.

**Child health indicators**				
**Variable**	**Follow-up survey** **(*****N*** **=** **119)**	**Baseline field survey** **(*****N*** **=** **102)**	**% change in coverage between follow-up and baseline field survey**	***p*** **-value**
**Immunization status**
**At birth**
BCG	95	96.1	−1.1	0.69
OPV 0	79	70.6	8.4	0.15
HBV 0	67.2	35.3	31.9	<0.0001
**At 1.5 months**
OPV 1	97.5	96.1	1.4	0.553
Pentavalent 1	98.3	96.1	2.2	0.307
**At 2.5 months**
OPV 2	98.3	94.1	4.2	0.095
Pentavalent 2	100	95.1	4.9	0.015
**At 3.5 months**
OPV 3	95	94.1	0.9	0.783
Pentavalent 3	96.6	94.1	2.5	0.369
**9 months**
MR/Measles	85.7	93.1	−7.4	0.77
Vitamin A	84.9	91.2	−6.3	0.154
Fully immunized	77.3	87.3	−10	0.056

*BCG, Bacillus calmette–guérin vaccine; OPV, Oral poliovirus vaccines; HBV, Hepatitis B vaccine; MR, Measles rubella vaccine*.

Pearson Chi-square tests were used to assess the association between the change in coverage of various child immunization services with the launch of the TeCHO+ programme. The improvement in coverage of HBV0 vaccination (67.2 vs. 35.3%, *p*-value < 0.0001) and Pentavalent 2 (100 vs. 95.1%, *p*-value = 0.015) were found to be statistically significant at 5% level of significance and 95% Confidence Interval.

#### Quality of Morbidity Indicators Among High-Risk Pregnant Women and Children

Quality and coverage of health services among high-risk women were evaluated. TeCHO+ programme has resulted in identifying high-risk women suffering from pregnancy-induced hypertension, severe maternal anemia or gestational diabetes, and the identification of high-risk children with low birth weight and severe acute malnutrition (Supplementary Tables 2, 3). During field validation, all these high-risk women and children were found, and these led us to believe that the quality of reporting has improved, although, without baseline estimates available, we have limited confidence to report on the improvement.

### Key Findings From Qualitative Inquiry

**Figure 4:**
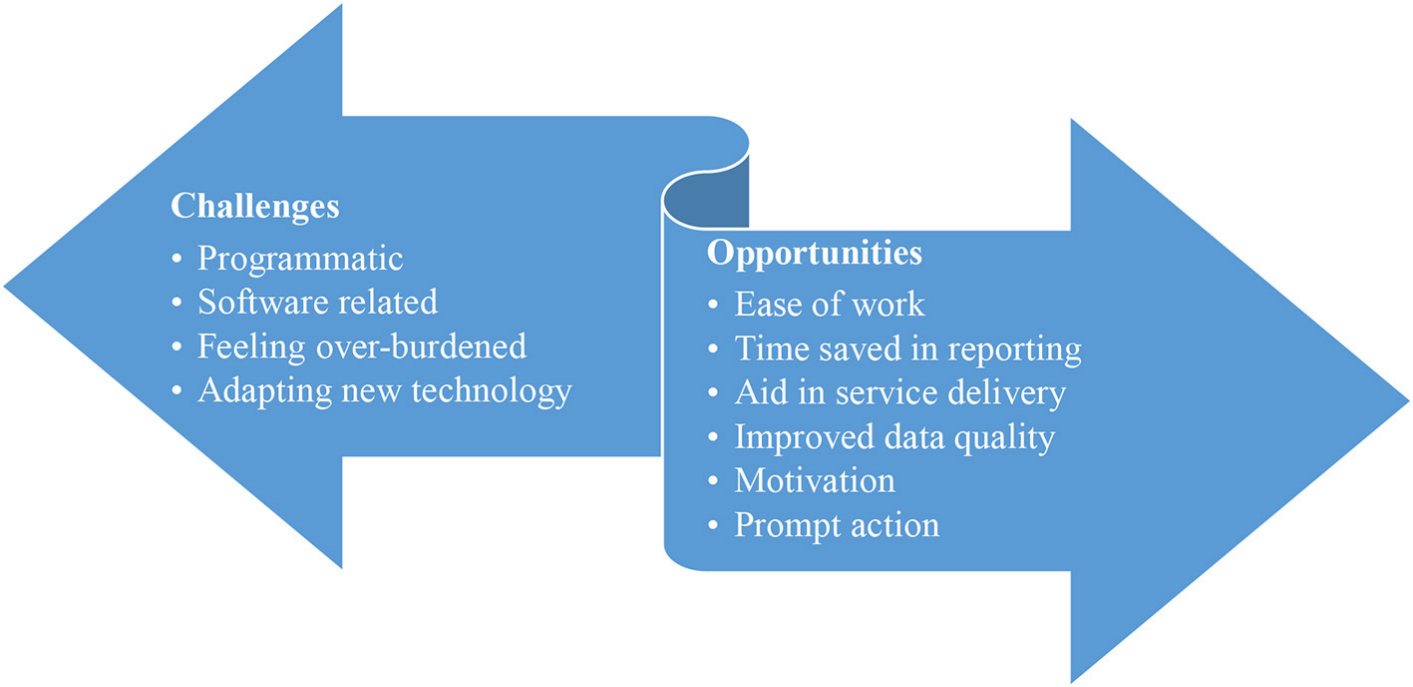
Challenges and opportunities of TeCHO+ implementation.

Findings from the qualitative inquiry are categorized into two broad categories: Challenges and Opportunities (Figure 4).

As shown in Figure 4, the category opportunities included sub-themes such as ease of work, time saved in reporting, aid service delivery, improved data quality, motivation, and prompt actions. Challenges primarily included programmatic, software related, feeling over-burdened and adapting new technology, many of which were reportedly addressed by the State team.

#### Challenges

Various challenges accompanied the statewide rollout of the TeCHO+ programme as described below.

##### Programmatic Challenges

Engaging field staff for the programme, acceptance of TeCHO+ and use of smart phones by ANM, mobilizing them for training and monitoring were crucial programmatic challenges. Due limited capacity to arrange in-person training for the field staff across the state, trainings were organized using satellite communication (SATCOM) infrastructure. However, most participant (especially ANM) did not find it beneficial. They preferred in-person training.

“I was reluctant for the TeCHO+ as I had never used smartphone in my life. Initially fellow team used to help me operate it. And gradually I learned the use of smart phone and operate TeCHO+. Now I am happy using TeCHO+. An ANM

##### Feeling Over-Burdened

Few ANMs (11%, *n* = 3) reported a high work burden as they maintained a manual register due to the fear of losing mobile data.

“Initially, ANMs were asked to maintain register along with data entry in TeCHO+ app. So, during that phase, many ANM felt burdened to manage data entry in both TeCHO+ app and physical register.” A Medical Officer

“We have no internet connectivity in my areas so had to operate offline. It was difficult operating TeCHO+ offline as many details of the beneficiaries were not accepted…[Thus] I had to maintain register which is burdonsome.” An ANM

##### Software Related Problems

About 38% (*n* = 15) of ANMs and MOs reported software related issues such as slow internet speed, frequent software hang ups, unavailability of editing or deleting options, difficulties in understanding medical terminology in English, issues in data entry.

“…we face problems while updating beneficiary information. Software asks for repeated entry on full-name of a beneficiary, date of birth, height, blood group, LMP, Tubal Ligation, date of CU-T insertion. It is so annoying that every time we update software, we have to fill basic information of beneficiaries.” An ANM

##### Adapting New Technology

More than half (55%, *n* = 16) participants (especially ANMs) were in their fifties and were first-time smartphone users. Although all participants readily accepted the new technology, they were facing difficulties in operating TeCHO+ app. Further, ANMs have also reported difficulties in coordinating with other ANMs regarding in/out migration of beneficiaries.

#### Opportunities

Study revealed six key sub-themes related to the theme-opportunity. These include, ease of work, time-saved in reporting, aid in service delivery, improved data quality, motivation and prompt action.

##### Ease of Work

Generally, TeCHO+ is perceived to be easier and effective by both ANM and Medical Officers (89%, *n* = 35) interviewed in this study. ANM stated that user-friendly application, use of regional language in the application, timely software update, and self-assessment of work are key strengths of the programme. An ANM said, “Identifying beneficiary from line list and locating beneficiary's house is easy through TeCHO+.” In addition, MO reported that the in-built supervisor's check has added value to the programme.

##### Time Saved in Reporting

One of the important points noted was saving time for micro-planning and preparing the monthly report as these get automatically generated based on data entered into the system. About 94% (*n* = 39) participants (both ANM & DEOs) felt that there was no need to spare extra time for micro-planning and preparing monthly report. On average, an ANM saved 1.7 h/day after the implementation of the TeCHO+ programme. DEO reported saving 1.5 h/day after introducing TeCHO+.

##### Aid in Service Delivery

About 70% of ANM (*n* = 22) have affirmed that the TeCHO+ programme has increased registration of early ANC, prevented duplication and false entry, improved coverage, and enabled timely service delivery through the high-risk alert system.

“Alerts for vaccination, the high-risk case helps us identify risk-cases early and enable us to provide timely services including referral” – ANM

##### Improved Data Quality

Users (59%, *n* = 23) have perceived TeCHO+ as a more reliable source of beneficiary data. A medical officer said, “…the data of TeCHO+ is more reliable due to close supervision and software mechanism*.”* Excerpts from few participants provide a glimpse of the ease of data management and quality of data reporting.

“Every day during log-in, we receive our work plan. If we make any error, the software does not allow us to enter. Thus, there are fewer chances of errors…” – ANM

“Report is auto-generated for ANM, for us [Medical Officer] as well as District, and State level officials. We get regular data updates from the software so that we can monitor and cross-check in the field.

##### Motivation

A strong supportive supervision mechanism through WhatsApp groups, helplines, and timely instructions from supervisors motivate the field team. About 68% (*n* = 20) felt motivated to use TeCHO+ application. An ANM expressed, “…receiving immediate solution for field problems over WhatsApp and voice calls is motivating.”

Instructions and reminders were shared through WhatsApp, and for urgent issues, voice calls were used to discuss and resolve. In addition, good works by ANMs were appreciated through WhatsApp, which built confidence and promoted peer-mentoring and encouraged many poor-performing ANMs to enhance their work.

##### Prompt Actions

Flexible and prompt solution-focused decisions from the state and district programme team prevented hindrances in implementation. Most ANMs (78%, *n* = 23) perceived that the district officials aptly addressed their field level issues immediately. Furthermore, the programme has decentralized actions at the district level to address field-level challenges locally. For example, the Dang district had cellular network problems. District team immediately allowed block team and ANMs to use cellular network which is locally available.

## Discussion

The study shows that there has been significant improvement in coverage of health services, quality of data reporting of various maternal and child high-risk conditions after the introduction of TeCHO+ software compared to the previous MCTS software. TeCHO+ software was incorporated with features such as real-time data entry, provision of on field supportive supervision, and helpline for immediate trouble-shooting to improve the quality of data reporting. In addition, features such as generation of daily micro-plan, real-time monitoring and counseling the beneficiaries targeted higher coverage of health services. The features of the TeCHO+ software thus addressed both supply-side and demand-side challenges to the service coverage. As per our knowledge, this is the first evaluation conducted by an independent agency of a mHealth programme implemented at scale in India, showing promising results.

mHealth has been an approach used for service delivery, including Communicable and Non-Communicable diseases, overall General Health and mainly for Reproductive and Child Health. Global studies on mHealth interventions showed significant improvements in maternal service utilization. The Wired-Mothers intervention of Lund et al. more than doubled the odds of a woman receiving four or more ANC visits (OR 2.39, 95% CI 1.03–5.55) (18). The pre-post intervention study of text message programme in Thailand also showed higher ANC attendance rates after reminders were sent *via* text messaging (ANC visits: OR 2.97, 95% CI 1.60–5.54) (19). In Sierra Leone, the mHealth intervention showed a positive net effect on facility-based service utilization for the following indicators: first and fourth ANC visit (0.7 and 11.3%-points, facility delivery (8.2%-14.6 points), and first, second and third PNC visit (10.1, 10.6 and 14.9%-points) (20). Oyeyemi and Wynn found a significantly higher facility utilization rate within the area in Nigeria taking part in a mHealth intervention (43.4% vs. 36.7%, *p* = 0.0001) (21). They defined facility utilization rate as the number of deliveries in a particular health facility to the number of ANC registrations in that same facility (22).

A systematic review of 14 studies conducted in 2017 (23) found mHealth interventions effective in improving antenatal care and postnatal care services. This review suggested that mHealth solutions can improve preventive maternal healthcare services and maternal outcomes. Few studies also reported improvement in health outcomes. Lund et al. (18) observed a significant effect on perinatal mortality in their study conducted in Zanzibar using mHealth intervention. Their mHealth intervention combined unidirectional text messaging and direct two-way communication in a free call voucher system to provide education on pregnancy, reminders for antenatal care visits and an emergency medical response system. They found a significant decrease in the perinatal mortality rate of 50% (OR 0.50, 95% CI 0.27 to 0.90) (18). The total perinatal mortality rate based on stillbirth and neonatal mortality was 27 per 1,000 births, 19 per 1,000 births in the intervention group compared to 36 per 1,000 births in the control group (18). Jareethum et al. assessed the effect of two educational text messages sent weekly in Thailand, found no differences for infant birth weight and preterm delivery (24).

Results similar to our study findings were observed in mHealth projects implemented in a controlled setting. The Cluster Randomized Controlled Trial of “ImTeCHO” application conducted in Jhagadia, Bharuch, reported the effectiveness of the intervention in averting malnutrition and maternal health and improving coverage of essential maternal newborn package of services. The increase in preventive service coverage due to the ImTeCHO reduces illness during pregnancy, after childbirth, and during the neonatal period (3). Further, important matters related to mechanisms of change and their link to the ANM efficacy and motivation were examined in the ImTeCHO trial that preceded the scale up and reported in detail in peer reviewed publications (20, 22). Similar findings were reported by the “Reducing Maternal and Newborn Deaths (ReMiND)” study team. Moreover, it recorded increased coverage of iron-folic acid supplementation, full ANC, >2 tetanus toxoid vaccination and ambulance usage in Kaushambi of Uttar Pradesh (4). A study done by Balakrishnan et al. highlights the use of mobile health applications to maintain a continuum of care for maternal and child health services in Bihar and reported improvement in eight major service delivery components viz. early registration of pregnant women, three antenatal visits, tetanus toxoid immunization of the mother, iron and folic acid tablet supply, institutional delivery, postnatal home visits and early initiation of breastfeeding in the intervention areas when compared to entire Bihar (18). Unfortunately, there is no published evidence regarding the evaluation of large mHealth programs at scale in India.

There are several probable reasons that might explain the results of the study. Earlier, data were maintained on paper registers by ANMs, later carried to data entry operators at PHC who would enter data in e-Mamta portal, usually after a few days of service provision. The state government decided to remove paper registers and e-Mamta software because all ANMs started using TeCHO+ within a year; this decision reduced time spent on data management. In contrast, data capture at the point and time of service by the service providers in the TeCHO+ software might have improved the data quality. Subsequently, reports generated from the TeCHO+ software based on good quality data became a single source of information and were extensively used by administrators for program monitoring at every level. TeCHO+ generated value for ANMs as well as administrators.

This study has some strengths. The study was done by an independent agency that was not involved in the TeCHO+ programme. Funding for the study was provided through an independent agency. The data used for this study was collected during household surveys instead of using program data from TeCHO+ software.

However, there were some limitations of this study. There was no control group because the TeCHO+ programme was implemented across the entire state of Gujarat at the same time. Therefore, the pre and post intervention study design used for the current study was the best possible option. The results regarding time saved were based on interviews with the health workers. Time series is a better method to estimate time saved. There is a scope for an in-depth assessment of the connective tissue of why something changed, motivation of ANMs over time, motivation, capacity and satisfaction. There is a need for further work to assess how could the changes have sustained beyond the initial phase.

## Conclusion

TeCHO+ programme can be a job aid for frontline health workers and show improvement in the coverage of full ANC, iron-folic acid, early initiation of breastfeeding and postnatal home visits, and quality of data reporting as it addressed both supply side as well as demand side challenges. Considering the influence of the technological intervention on quality and efficiency of service delivery and health care workforce efficiency, this mHealth application should be considered for scale-up in other states. Its effectiveness might be evaluated using more robust study designs during further scale-up.

## Data Availability Statement

The raw data supporting the conclusions of this article will be made available by the authors, without undue reservation.

## Ethics Statement

The studies involving human participants were reviewed and approved by Institutional Ethics Committee of Indian Institute of Public Health Gandhinagar. The patients/participants provided their written informed consent to participate in this study.

## Author Contributions

SS conceptualized the study, led the study, analyzed, and drafted the manuscript. ZQ contributed in the study design and analysis plan. Both authors contributed to the article and approved the submitted version.

## Funding

This study was supported through the Health Technology Assessment Regional Resource Centre at the Indian Institute of Public Health Gandhinagar, funded through the Department of Health Research, Government of India.

## Author Disclaimer

The expressed views and opinions do not necessarily express the policies of the Department of Health Research and the Health and Family Welfare Department, Gujarat.

## Conflict of Interest

The authors declare that the research was conducted in the absence of any commercial or financial relationships that could be construed as a potential conflict of interest.

## Publisher's Note

All claims expressed in this article are solely those of the authors and do not necessarily represent those of their affiliated organizations, or those of the publisher, the editors and the reviewers. Any product that may be evaluated in this article, or claim that may be made by its manufacturer, is not guaranteed or endorsed by the publisher.
