# Supplementary material for: Does Digitally Enabling Frontline Health Workers Improve Coverage and Quality of Maternal and Child Health Services? Findings From a Mixed Methods Evaluation of TECHO+ in Gujarat
Source: Front Public Health. 2022 Jul 22;10:856561. doi: 10.3389/fpubh.2022.856561 (PMC9363132; doi:10.3389/fpubh.2022.856561)
Supplement: Supplementary file 1 [file Data_Sheet_1.pdf]

Supplementary File

Supplementary Table 1: List of Indicator for evaluation

Indicators
**Maternal Care**
Identification of Severe Maternal Anaemia
Mothers who received full antenatal care (%)
Mothers who received full postnatal care (%)
Institutional births (%)
**Morbidity Management among Mothers**
Identification of Severe Maternal Anaemia
Identification of Pregnancy Induced Hypertension and Management
**Children Care and immunizations**
Referral of Children to NRC/ CMTC/ CMAM (%)
Children age 12-23 months fully immunized (BCG, measles, and 3 doses each of polio and DPT) (%)
**Morbidity Management Among Children**
Identification of lbw (less than 2.5 kg) and its management
Identification of SAM (%)
**Feeding Practices**
Early Initiation of Breast Feeding(%)

Supplementary Table 2: Quality Assessment of indicators among High Risk Mother

High Risk Mother Health Indicators			
**Pregnancy Induced Hypertension (PIH)**			
**Variables**	**TeCHO+ (N=9)**	**On Field out of TeCHO+**	**Quality of Data reporting in TeCHO+/ Concordance**
Identification of PIH	3	3	100%
**Severe Maternal Anemia (SMA)**			
**Variables**	**TeCHO+ (N=39)**	**On Field out of TeCHO+**	**Quality of Data reporting in TeCHO+/ Concordance**
Identification of SMA	13	13	100%
**Gestational Diabetes (GD)**			
**Variables**	**TeCHO+ (N=10)**	**On Field out of TeCHO+**	**Quality of Data reporting in TeCHO+/ Concordance**
Identification of GD	3	3	100%

Supplementary Table 3: Quality Assessment of indicators among High Risk Children

High Risk Child Health Indicators			
**Low Birth Weight**			
**Variables**	**TeCHO+ (N=81)**	**On Field out of TeCHO+**	**Quality of Data reporting in TeCHO+/ Concordance**
Child identified as LBW	60	60	100%
**Severe Acute Malnutrition**			
**Variables**	**TeCHO+ (N=60)**	**On Field out of TeCHO+**	**Quality of Data reporting in TeCHO+/ Concordance**
Child identified as SAM	50	50	100%
